# Targeting the Metabolic Rewiring in Pancreatic Cancer and Its Tumor Microenvironment

**DOI:** 10.3390/cancers14184351

**Published:** 2022-09-07

**Authors:** Keisuke Yamamoto, Dosuke Iwadate, Hiroyuki Kato, Yousuke Nakai, Keisuke Tateishi, Mitsuhiro Fujishiro

**Affiliations:** 1 Department of Gastroenterology, Graduate School of Medicine, The University of Tokyo, 7-3-1 Hongo, Bunkyo-ku 113-8655, Tokyo, Japan; 2 Division of Gastroenterology, Department of Internal Medicine, St. Marianna University School of Medicine, 2-16-1 Sugao, Kawasaki City 216-8511, Kanagawa, Japan

**Keywords:** pancreatic ductal adenocarcinoma, KRAS, tumor microenvironment, metabolic rewiring, anabolic glucose metabolism, glycolysis, pentose phosphate pathway, hexosamine biosynthesis pathway, glutamine metabolism, macropinocytosis, autophagy, lysosome, immune checkpoint blockade, immune evasion, MHC-I, serine biosynthesis pathway

## Abstract

**Simple Summary:**

Pancreatic ductal adenocarcinoma (PDAC) is characterized by its unique metabolic properties that are established both in a cell-intrinsic and -extrinsic manner, dictated by oncogenic KRAS signaling and shaped in close interaction with the host cells in the tumor microenvironment. Understanding these properties and the underlying mechanisms may reveal novel vulnerabilities that can be therapeutically targeted to improve the patient outcomes and overcome treatment resistance. This review summarizes the mechanisms by which PDAC cells utilize limited nutrients to maximize their growth and obtain nutrients from inside and outside the cells to thrive in a nutrient-scarce microenvironment, with a particular focus on the roles of autophagy in the pathogenesis of PDAC.

**Abstract:**

Pancreatic ductal adenocarcinoma (PDAC) is an aggressive malignancy with only a few effective therapeutic options. A characteristic feature of PDAC is its unique tumor microenvironment (TME), termed desmoplasia, which shows extensive fibrosis and extracellular matrix deposition, generating highly hypoxic and nutrient-deprived conditions within the tumor. To thrive in this harsh TME, PDAC undergoes extensive metabolic rewiring that includes the altered use of glucose and glutamine, constitutive activation of autophagy-lysosomal pathways, and nutrient acquisition from host cells in the TME. Notably, these properties support PDAC metabolism and mediate therapeutic resistance, including immune suppression. A deeper understanding of the unique metabolic properties of PDAC and its TME may aid in the development of novel therapeutic strategies against this deadly disease.

## 1. Introduction

Pancreatic ductal adenocarcinoma (PDAC) is a deadly disease with a 5-year survival rate of approximately 10%, and is among the worst of all cancer types [1,2]. With a consistently increasing number of cases, PDAC is estimated to become the second leading cause of cancer-related deaths in the United States by 2030 [3]. The poor prognosis of PDAC may be attributed to the difficulty in early diagnosis, almost inevitable recurrence after surgery, limited treatment options, and therapeutic resistance. 

Early detection of PDAC is extremely difficult because of the lack of specific symptoms, reliable biomarkers, and effective screening methods. Thus, 80–90% of patients present with advanced disease, who are not eligible for surgical resection. For eligible patients, surgery is the only treatment with curative intent, although the recurrence rate after resection is as high as 80%, with most patients succumbing to disease recurrence and only about 10–20% patients showing a 5-year survival. Chemotherapy remains the main treatment option for patients with unresectable disease [4].

PDAC is notoriously treatment resistant. Even though recent developments of potent combination regimens, such as FOLFIRINOX or gemcitabine plus nab-paclitaxel (GnP), have remarkably improved the patient prognosis [5,6], the outcomes remain unsatisfactory. Moreover, compared to other malignancies, effective treatment regimens for PDAC are still lacking because of its extreme resistance to currently available therapeutic options. For example, although the discovery of immune checkpoint blockade (ICB) therapies has drastically improved the patient prognosis in several types of cancer, the vast majority of patients with PDAC are unresponsive to ICB [7,8,9], apart from some patients (1–2%) with high microsatellite instability (MSI-high) due to mutations in DNA mismatch repair pathways (MLH1, MSH2, MSH6, and PMS2), which show significant responses to programmed death-1 (PD-1) blockade [10,11,12,13]. 

The most frequently mutated genes in PDAC are the oncogene *KRAS* and the tumor suppressor genes, tumor protein p53 (TP53), SMAD family member 4 (SMAD4), and cyclin-dependent kinase inhibitor 2A (CDKN2A), which occur in >90%, 60–70%, >50%, and >50% of cases, respectively [14]. As these major mutations in PDAC are still undruggable, only a subset of patients with PDAC benefit from molecular-targeted therapies based on these genetic mutations. These include patients with mutations in DNA damage repair (DDR) pathway genes (4–7%), such as BRCA1/2, who are responsive to platinum-based chemotherapy [15,16] or maintenance therapy with the poly-ADP ribose polymerase (PARP) inhibitor, olaparib, after disease stabilization with platinum-based therapy (POLO study) [17]. Patients with KRAS^G12C^ mutation (1% in patients with PDAC) [18] are sensitive to KRAS^G12C^ inhibitors [19,20,21,22], and those with the neurotrophic receptor tyrosine kinase (*NTRK*) gene fusion (0.4%) are responsive to entrectinib [23]. Thus, it is vital to identify the vulnerabilities in this deadly disease to provide more therapeutic opportunities and aid in drug development.

One key feature of PDAC is its dense fibrotic tumor stroma, termed desmoplasia [24], which plays pivotal roles in various aspects of PDAC biology, including therapeutic resistance and unique metabolic properties. The tumor microenvironment (TME) of PDAC tumors is composed of abundant stromal cells and extracellular matrix (ECM) components, such as collagen and hyaluronic acid (HA) [25]. 

Stromal cells include cancer-associated fibroblasts (CAFs), endothelial cells, nerve cells, and various immune cells. Of note, most of the immune cells in the PDAC TME are immune suppressive or pro-tumor, such as myeloid-derived suppressor cells (MDSCs) or tumor-associated macrophages (TAMs) [26], which create an immune suppressive microenvironment that hinders efficient anti-tumor immune response, resulting in resistance to ICB [27]. Additionally, stromal cells confer treatment resistance to PDAC cells or promote PDAC growth by supporting their metabolism [28].

ECM deposition, which sometimes accounts for the largest proportion of PDAC tumors, is not a mere scaffold and plays complex roles in PDAC biology. For example, HA, a major component of the ECM in PDAC tumors, is a hydrophilic glycosaminoglycan that attracts and retains water in the tumor bed. This leads to an elevated interstitial fluid pressure, collapsing the blood vessels and blocking the efficient exchange of oxygen, nutrients, and metabolic wastes as well as the diffusion of chemotherapy drugs [29,30]. Consequently, dense ECM deposition creates a metabolically harsh environment for PDAC, where oxygen and nutrients are scarce and metabolic waste is accumulated. Indeed, studies using human and mouse PDAC tumors have shown that PDAC is among the most hypoxic [31,32] and nutrient-deprived [33,34] tumors of all cancer types. 

PDAC cells reprogram their metabolism to survive and grow in metabolically challenging TME [28,35,36,37,38,39,40,41]. This includes the rewiring of central carbon metabolism, which enables distinct utilization of glucose and glutamine, increased nutrient scavenging from outside and inside the cells (macropinocytosis and autophagy), and acquisition of nutrients from non-cancerous cells in the TME. In this review, we discuss the unique metabolic properties of PDAC cells and surrounding host cells as well as the metabolic crosstalk between PDAC and stromal cells. A deeper understanding of these properties may help in identifying the novel vulnerabilities of this deadly disease and facilitate the development of new therapeutic strategies.

## 2. Glucose and Glutamine Metabolism in PDAC

**Figure 1:**
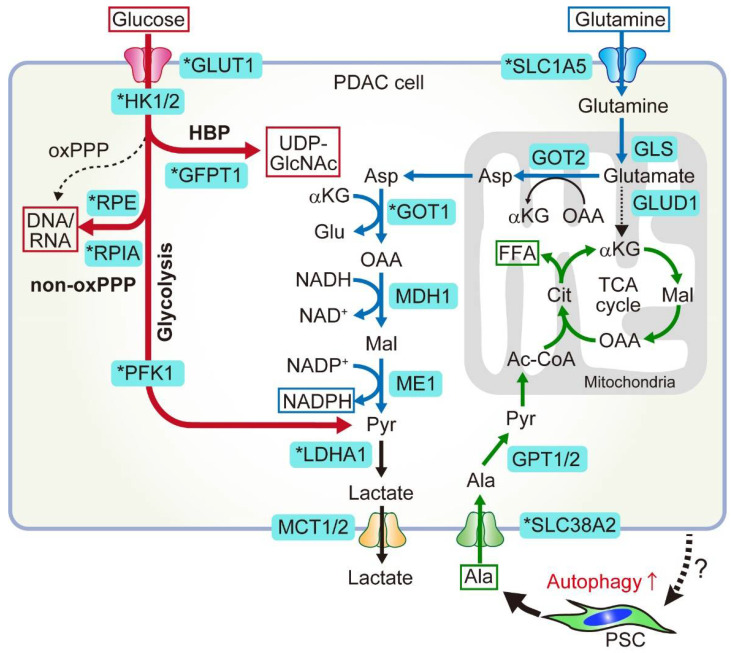
Central carbon metabolism in pancreatic ductal adenocarcinoma (PDAC) is rewired in a cell-autonomous manner by oncogenic KRAS signaling and in a cell-extrinsic manner via interaction with host stromal cells. Key enzymes that are upregulated by oncogenic KRAS are indicated with an asterisk. (Glucose metabolism, shown in red) Glucose is preferentially used for anabolic processes (anabolic glucose metabolism) in PDAC. This is mediated by the upregulation of key enzymes involved in those processes. Glucose transporter 1 (GLUT1) upregulation increases the glucose uptake, which fuels glycolysis and branching anabolic pathways. These include the hexosamine biosynthesis pathway (HBP), which produces precursors for glycosylation, and the non-oxidative pentose phosphate pathway (non-oxPPP), which produces ribose for nucleotide biosynthesis. (Glutamine metabolism, shown in blue) Glutamine is a key substrate that is used to fuel the tricarboxylic acid (TCA) cycle and maintain redox balance in PDAC, which is mediated by a pathway distinct from all other types of cancers. (PSC-derived alanine, shown in green) As shown above, glucose and glutamine are primarily used for anabolic pathways and redox maintenance in PDAC. To supplement the TCA cycle, PDAC cells utilize alanine that is released from pancreatic stellate cells (PSCs) in the tumor microenvironment (TME). In response to stimuli from PDAC (unknown), PSCs secrete alanine through SLC1A4 in an autophagy-dependent manner. PDAC cells upregulate SLC38A2 to uptake PSC-derived alanine, which then fuels the TCA cycle and is used to produce macromolecules, including free fatty acids (FFA). Ac-CoA, acetyl coenzyme A; Asp, asparagine; αKG, α-ketoglutarate; Cit, citrate; Mal, malate; OAA, oxaloacetate. Figure is adapted from reference [41], the use of which is licensed under a Creative Commons Attribution 4.0 International license https://creativecommons.org/licenses/by/4.0/.

Altered metabolism has long been recognized as a hallmark of cancer [42]. This holds true for PDAC, where its metabolism is rewired either in a cell-intrinsic manner under genetic alterations or in a cell-extrinsic manner, triggered and shaped by extracellular cues, such as environmental changes or interaction with stromal cells in the tumor. Metabolic alterations induced by oncogenic KRAS include anabolic glucose metabolism, which provides precursors for biomass production for cell proliferation (Figure 1, red), altered glutamine metabolism that helps maintain cellular redox homeostasis (Figure 1, blue), and upregulated scavenging pathways to fulfil increased nutrient demands. PDAC metabolism is also shaped and supported by metabolites or cytokines secreted from the host microenvironmental cells.

### 2.1. Anabolic Glucose Metabolism

Glucose is an important energy source. The glycolytic pathway is a sequence of reactions that uses glucose as a substrate to produce adenosine triphosphate (ATP), an energy molecule, and two molecules of pyruvate. In non-proliferative cells, pyruvate typically enters the mitochondria, where it fuels the tricarboxylic acid (TCA) cycle and oxidative phosphorylation to generate multiple ATP molecules [43]. In proliferative cells, such as cancer cells, glucose is also important as a carbon source to produce building blocks, as most glycolysis intermediates are substrates of multiple anabolic pathways that are branched from the glycolytic pathway. Glucose is one of the scarcest nutrients in PDAC tumors [33,34], and to cope with its paucity, PDAC cells enhance glucose uptake and increase the glucose flux into glycolysis. This results in the increased production of glycolytic intermediates, which are shunted into anabolic pathways, such as the hexosamine biosynthesis pathway (HBP), non-oxidative branch of the pentose phosphate pathway (PPP), serine synthesis pathway, and subsequent one-carbon metabolism pathway. These metabolic alterations are driven by oncogenic KRAS, which transcriptionally upregulates key metabolic genes. These include the glucose transporter 1 (GLUT1), which promotes glucose uptake; the enzymes hexokinase 1/2 (HK1/2), phosphofructokinase 1 (PFK1), and lactate dehydrogenase A (LDHA), all of which enhance glycolytic flux; glutamine-fructose-6-phosphate transaminase 1 (GFPT1), a rate-limiting enzyme for HBP; and ribulose-5-phosphate-3-epimerase (RPE) and ribose-5-phosphate isomerase A (RPIA), which are rate-limiting enzymes for non-oxidative PPP (Figure 1). 

#### 2.1.1. HBP

The HBP pathway produces uridine diphosphate N-acetyl glucosamine (UDP-GlcNac), a major substrate for protein glycosylation. Importantly, knockdown of Gfpt1, the rate-limiting enzyme for HBP in PDAC cells, reduced total O-linked N-acetylglucosamine (O-GlcNAc) levels and tumor growth both in vitro and in vivo [35], supporting the essential role of HBP and protein glycosylation in PDAC tumor maintenance and growth.

#### 2.1.2. Non-Oxidative PPP

PPP is a side branch of glycolysis that produces NADPH and ribose-5-phospate (R5P). NADPH is an important reducing equivalent for redox control or biosynthesis of other molecules, including lipids, whereas R5P is an essential substrate for nucleotide biosynthesis. PPP is composed of two arms: the oxidative and non-oxidative arms. Oxidative PPP (oxPPP) generates NADPH, whereas non-oxidative PPP (non-oxPPP) generates nucleic acids. In PDAC cells, oncogenic KRAS preferentially increases the flux through non-oxPPP to promote DNA/RNA synthesis without affecting the oxPPP that generates antioxidant NADPH. Accordingly, glucose deprivation has only minimal effects on ROS levels and significantly impairs PDAC growth, suggesting that glucose is not primarily used to maintain the cellular redox balance. 

This preferential activation of non-oxPPP relies on KRAS-mediated transcriptional upregulation of the two key enzymes for non-oxidative PPP, RPE, and RPIA. Importantly, inhibition of non-oxPPP through knockdown of RPE and RPIA significantly reduced the flux of ^14^C_1_-labeled glucose into DNA/RNA and impaired PDAC growth both in vitro (particularly under the low glucose condition that recapitulates PDAC tumor) and in vivo, supporting the essential role of non-oxPPP in PDAC pathogenesis. Moreover, enhanced glycolysis and glucose flux into the non-oxPPP also contributes to resistance to gemcitabine (GEM), a deoxycytidine analog that terminates DNA synthesis and a mainstay chemotherapy drug for PDAC, by increasing de novo synthesis of pyrimidine derivatives, including deoxycytidine triphosphate (dCTP), which competitively attenuates the efficacy of GEM [44]. Thus, rewired glucose metabolism not only facilitates PDAC proliferation under a glucose-deprived TME but also affords therapeutic resistance to PDAC.

### 2.2. Non-Canonical Glutamine Metabolism

Mitochondria are intracellular organelles that provide both energy and anabolic substrates for cell proliferation and homeostasis through TCA cycle activity and oxidative phosphorylation (OXPHOS). The TCA cycle uses pyruvate as a major substrate, but also uses other substrates, such as fatty acids and amino acids, including glutamine and alanine, depending on their availability. Glutamine is the most abundant amino acid in blood. In addition to fueling the TCA cycle, glutamine is also used to produce other amino acids, nucleic acid precursors, such as pyrimidines and purines, and antioxidants, such as glutathione (GSH) and NADPH, thereby fueling multiple biosynthetic pathways and maintaining the cellular redox balance [39] (Figure 1).

#### 2.2.1. Canonical and Non-Canonical Glutamine Metabolism

Given its multifaceted roles and abundance in the blood, it is not surprising that many cancers use glutamine to supplement the relative shortage of glucose or maintain cellular redox. Indeed, enzymes involved in glutamine metabolism have been targets of drug development. For example, many cancers use glutamine to produce α-ketoglutarate, which enters the TCA cycle and generates intermediate molecules, such as proteins, lipids, and nucleic acids, for biomass production. Canonical glutamine metabolism depends on glutamate dehydrogenase (GLUD1) (Figure 1). In contrast, PDAC cells use glutamine through a non-canonical pathway that is critical for maintaining redox homeostasis [45]. In PDAC cells, glutamine-derived glutamate in the mitochondria is converted to α-ketoglutarate and aspartate through the activity of mitochondrial aspartate transaminase (GOT2) rather than GLUD1. Aspartate is then transported from the mitochondria to the cytoplasm, where it undergoes a series of reactions to generate the key antioxidant NADPH and to maintain redox homeostasis in PDAC. The enzymes involved in these reactions include cytosolic aspartate aminotransferase (GOT1), malate dehydrogenase (MDH1), and malic enzyme (ME). Genetic inhibition of these key enzymes impairs PDAC growth both in vitro and in vivo. This non-canonical glutamine metabolism is facilitated by oncogenic KRAS, which suppresses GLUD1 and upregulates GOT1 expression in PDAC cells. Notably, GOT1 inhibition impairs the growth of PDAC cells but does not affect the growth of normal pancreatic duct cells, providing a good rationale for targeting GOT1 as a therapeutic strategy against PDAC [45]. Accordingly, GOT1 inhibition-mediated impairment of redox balance synergizes with radiotherapy, a pro-oxidant treatment, in PDAC mouse models [46].

#### 2.2.2. Targeting Glutamine Metabolism

Based on its critical role, several attempts have been made to target glutamine metabolism in PDAC.

- Glutaminase inhibitors

Glutaminase (GLS) is an enzyme that converts glutamine to glutamate, the first step in glutamine metabolism that is commonly required for both canonical and non-canonical pathways (Figure 1, blue); hence, it is an attractive target for drug development. However, inhibition of GLS with a clinical-grade inhibitor CB-839 failed to show efficacy against PDAC, mainly due to the rapid development of resistance that is mediated by compensatory metabolic changes [47]. This study highlights the robustness of PDAC cells against metabolic perturbations and the importance of developing combined approaches that enable efficient blockage of adaptation mechanisms. Notably, prolonged treatment with CB-839 increased metastasis in a PDAC mouse model [47]. Mechanistically, a recent study demonstrated that glutamine deprivation induces epithelial-to-mesenchymal transition (EMT) [48], a process implicated in various aspects of cancer cells, including metastasis formation, raising concerns about glutamine metabolism inhibition as a therapeutic means and highlighting the need for a thorough understanding of its impacts on PDAC progression.

- Glutamine antagonist

6-diazo-5-oxo-L-norleucine (DON) is a pan-glutamine antagonist that inhibits all metabolic reactions utilizing glutamine. Although DON failed to show efficacy against cancers in clinical trials, mainly due to severe gastrointestinal toxicities [49], DON treatment showed anti-tumor effects and synergized with anti-PD1 treatment in a PDAC mouse model [50]. Notably, this immune modulatory effect of glutamine metabolism blockade was also demonstrated in studies using a newly developed prodrug of DON, JHU083, against colon cancer, lymphoma, and melanoma, in which JHU083 treatment showed synergistic effects with immune checkpoint blockade therapy [51,52]. Further studies are warranted to pave the way for clinical use of these inhibitors.

## 3. Nutrient Scavenging Pathways in PDAC

**Figure 2:**
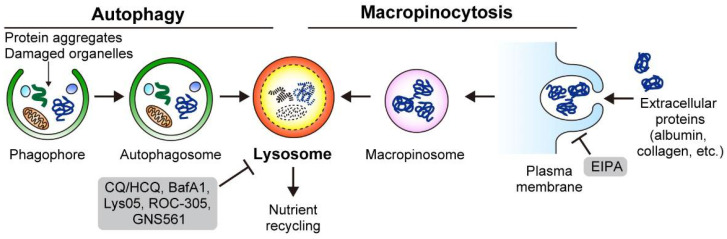
To thrive in the nutrient-deprived TME, PDAC relies on autophagy and macropinocytosis for intracellular nutrient recycling and extracellular nutrient scavenging. Autophagy targets the intracellular constituents, such as protein aggregates and damaged organelles, while macropinocytosis engulfs the extracellular nutrients via protrusion of the plasma membrane. Both autophagy and macropinocytosis depend on the lysosome for the degradation of their cargos and recycling of nutrients. Inhibitors are shown in gray frames. Figure 2 is adapted from reference [41], the use of which is licensed under a Creative Commons Attribution 4.0 International license https://creativecommons.org/licenses/by/4.0/.

To survive in a nutrient-scarce microenvironment, PDAC depends on two lysosomal scavenging pathways: autophagy and macropinocytosis. While autophagy targets and recycles intracellular constituents, macropinocytosis captures extracellular materials to acquire nutrients (Figure 2). Both pathways support PDAC cell metabolism by satisfying the metabolic demands.

### 3.1. Macropinocytosis

#### 3.1.1. PDAC Cells Use Macropinocytosis to Scavenge Extracellular Proteins and Lipids

Macropinocytosis is an evolutionarily conserved endocytic process, whereby extracellular fluid and proteins are internalized and degraded through large vesicles called macropinosomes. Commisso et al. showed that RAS-mutant cancers, including PDAC, exhibit enhanced macropinocytosis, thereby increasing the uptake of extracellular proteins, such as serum albumin, which serves as an important amino acid source for central carbon metabolism. Thus, macropinocytosis supports PDAC growth under nutrient-limiting conditions, especially under glutamine-starved conditions [53]. Indeed, pharmacological inhibition of macropinocytosis by 5-[N-ethyl-N-isopropyl] amiloride (EIPA), a Na^+^/H^+^ exchanger (NHE) inhibitor, reduced PDAC proliferation both in vitro and in vivo [53] (Figure 2). This initial finding was further validated in studies using surgically resected human PDAC tumors and murine PDAC mouse models [33,54], highlighting the importance of macropinocytosis as a clinically relevant target in PDAC. 

#### 3.1.2. PDAC Cells Feed on ECM via Macropinocytosis

Which substrates are captured and utilized by PDAC via macropinocytosis? As mentioned previously, PDAC tumors have excessive ECM deposition, which hampers the efficient delivery of nutrients to PDAC cells and creates a nutrient-austere microenvironment. Recent studies showed that PDAC cells feed on collagen and HA, two most abundant ECM components, via macropinocytosis [55,56]. Olivares et al. showed that PDAC cells take up collagen fragments through macropinocytosis-dependent and macropinocytosis-independent mechanisms. The collagen-derived proline then fuels TCA cycle metabolism, promoting survival and proliferation of PDAC cells under nutrient-depleted conditions. Indeed, the genetic knockout of the proline catabolic enzyme PRODH1, which is overexpressed in PDAC cells, leads to a significant reduction in tumor growth in vivo [55]. 

As mentioned previously, PDAC cells show increased glycosylation precursor biogenesis through HBP, which is mediated by upregulation of the rate-limiting enzyme glutamine-fructose 6-phosphate aminotransferase 1 (GFAT1) [35]. Interestingly, Kim et al. reported that GFAT1 is required for PDAC cell proliferation in vitro but is unexpectedly dispensable for tumor growth in vivo. Mechanistically, PDAC cells take up HA into the TME via macropinocytosis. Composed of GlcNAc and glucuronic acid sugars, HA is used by PDAC cells as an alternative source of GlcNAc to refill the glycosylation pool, thus bypassing the effect of the GFAT1 knockout. Taken together, these results demonstrate that macropinocytosis allows PDAC cells to utilize the ECM as an alternative nutrient source under nutrient-limited conditions, enabling the metabolic flexibility of PDAC cells.

#### 3.1.3. Mechanisms for Enhanced Macropinocytosis

The regulators of enhanced macropinocytosis in cancer are an active area of research. Recent studies have identified multiple signalling pathways and molecules as key regulators of macropinocytosis. Lee et al. used human patient-derived xenografts and demonstrated that glutamine is depleted in the tumor core, where macropinocytosis is activated through EGFR signaling [57]. Ramirez et al. used a short-interfering RNA screen to identify vacuolar ATPase (V-ATPase) as a critical effector of *RAS*-driven macropinocytosis [58]. Yao et al. performed a functional proteomic screen in a doxycycline-inducible KRAS mouse model of PDAC (iKRAS) [35] and identified syndecan 1 as a novel effector of macropinocytosis in PDAC [59]. 

### 3.2. Autophagy

Macroautophagy (hereafter autophagy) is a well-conserved intracellular degradation pathway. One of the key features of PDAC is its constitutively active autophagy, which enables sustained PDAC growth through multiple mechanisms, including nutrient recycling, immune evasion, and treatment resistance. Thus, autophagy represents an attractive therapeutic target for PDAC, and multiple treatment regimens that include autophagy inhibition are currently being tested in clinical settings.

#### 3.2.1. Basics of Autophagy

Autophagy is a catabolic process in which cellular components are targeted for lysosomal degradation to recycle nutrients or maintain cellular homeostasis [60,61,62,63]. Autophagy captures its substrates in a double-membrane vesicle called an autophagosome, which subsequently fuses with the lysosome, a single-membrane organelle filled with acidic enzymes, to form an autolysosome. Autolysosome contents are digested and yield nutrients, such as amino acids, fatty acids, sugars, and nucleic acids, which are recycled back to the cytosol and used for various bioenergetic and anabolic processes, facilitating the survival of cells under nutrient-limited or nutrient-stressed conditions. Autophagy also contributes to the maintenance of cellular homeostasis by removing toxic or harmful components, such as protein aggregates, damaged organelles, or signaling molecules. Notably, these large molecules or organelles cannot be removed by the ubiquitin-proteasome system (UPS), which is another major intracellular degradation pathway. 

Autophagy was originally thought to be a non-selective process in which its targets are randomly captured and degraded. This process, known as bulk autophagy or general autophagy, mainly contributes to nutrient recycling under nutrient-deprived or nutrient-stressed conditions. However, later studies have shown that autophagy can also target and degrade specific substrates, such as protein aggregates or damaged organelles, to maintain cellular fitness. This process, termed selective autophagy, is enabled with the help of autophagy cargo receptor proteins, which bind to both autophagy substrates and LC3-II on autophagosomes, facilitating the trafficking and degradation of their targets. In general, selective autophagy is mediated by basal autophagy, which is constitutively activated at low levels to maintain cellular homeostasis, whereas bulk autophagy is acutely induced in response to various stresses, such as starvation, hypoxia, and low pH in the TME [64,65]. Importantly, both bulk and selective autophagy promote PDAC progression via multiple mechanisms.

#### 3.2.2. Molecular Mechanisms of Autophagy

**Figure 3:**
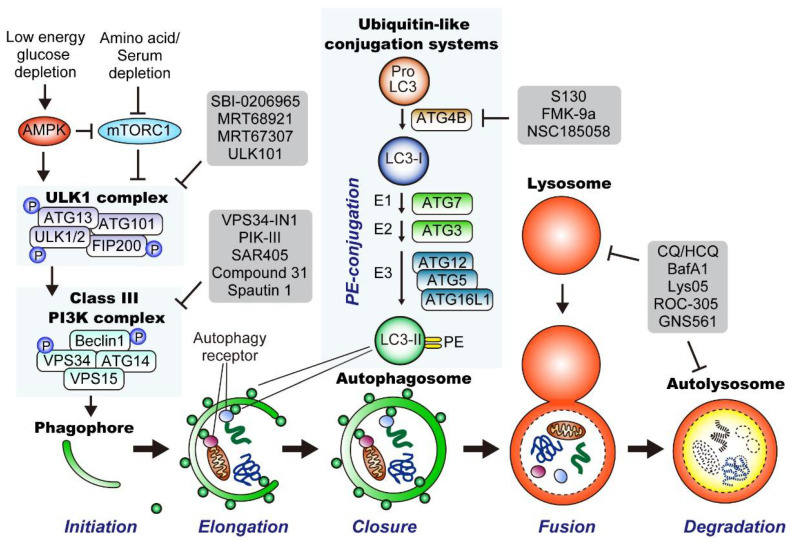
Overview of the general autophagy pathway in mammalian cells. (Bottom) Autophagy can be divided into five major steps: (1) Initiation and nucleation of the double-membrane phagophore, (2) elongation, (3) closure of the phagophore to form the autophagosome, (4) autophagosome-lysosome fusion; and (5) lysosomal degradation and nutrient recycling. (Top left) Upon activation by AMP-activated protein kinase (AMPK), the unc-51-like autophagy activating kinase 1 (ULK1) complex (also known as the preinitiation complex) initiates autophagy by activating the class III phosphatidylinositol 3-kinase (PI3K) complex (also known as the initiation complex) via phosphorylation of Beclin1 and VPS34. Thereafter, the activated class III PI3K complex generates phosphatidylinositol 3-phosphate (PI3P) at the site of nucleation of the phagophore from ER, leading to the binding of PI3P-binding proteins, such as WIPI proteins, that are required for phagophore maturation. (Top middle) ProLC3 is cleaved by ATG4B and to produce LC3-I. Thereafter, LC3-I is conjugated to phosphatidylethanolamine (PE) with a series of ubiquitin-like conjugation reactions that include the E1 ligase (ATG7), E2 ligase (ATG3), and the E3 ligase complex that is composed of ATG12, ATG5, and ATG16L. PE-conjugated LC3, termed LC3-II, is inserted into the phagophore membranes, where LC3-II promotes phagophore elongation and closure. LC3B-II also works as a docking site of autophagy receptor proteins, facilitating the trafficking of their cargos to the autophagosome, a process known as selective autophagy. Multiple inhibitors have been developed so far, which are shown in gray frames. ULK, Unc-51-like kinase; PI3K, phosphatidylinositol 3-kinase; PI3P, phosphatidylinositol 3-phosphate; ER, endoplasmic reticulum; WIPI, WD-repeat protein interacting with phosphoinositide; mTORC1, mammalian target of rapamycin complex 1; AMPK, 5′ AMP-activated protein kinase; PE, phosphatidylethanolamine. Figure 3 is adapted from reference [41], the use of which is licensed under a Creative Commons Attribution 4.0 International license https://creativecommons.org/licenses/by/4.0/.

Autophagy is a complicated multistep process tightly regulated by the coordinated action of more than 30 autophagy-related (ATG) proteins. Autophagy can be divided into five steps: (1) initiation and nucleation of the double-membrane phagophore (also termed the isolation membrane), (2) elongation, (3) closure of the phagophore to form the autophagosome, (4) autophagosome-lysosome fusion, and (5) lysosomal degradation and nutrient recycling (Figure 3). Below, we take a closer look at each step.

##### Initiation of Autophagy

Autophagy is induced in response to various stressors, including starvation, hypoxia, low pH, DNA damage, organelle damage, endoplasmic reticulum (ER) stress, and pathogen infection [62]. Among these, starvation has been the most extensively studied inducer of autophagy. The unc-51-like autophagy activating kinase 1 (ULK1) complex plays a central role in regulating autophagy induction by integrating signals from two major nutritional sensors: AMP-activated protein kinase (AMPK), an energy sensor protein that detects a reduction in cellular ATP levels [66], and mechanistic target of rapamycin complex 1 (mTORC1), a master regulator of cell growth that senses nutrient availability [67]. Under nutrient-rich conditions, mTORC1 is active and promotes cellular anabolism, while inhibiting catabolic processes, including autophagy. Activated mTORC1 blocks autophagy induction by suppressing the ULK1 complex via the phosphorylation of ULK1/2 or ATG13 [68]. In contrast, upon starvation and low cellular ATP levels, AMPK is activated, while mTOCR1 is inactivated, leading to the activation of the ULK1 complex and subsequent autophagy induction [66]. 

##### Generation of Phagophores

Upon activation, the ULK1 complex (also known as the pre-initiation complex) activates the class III phosphatidylinositol 3-kinase (PI3K) complex (also known as the initiation complex) through phosphorylation of Beclin1 and VPS34. The activated Class III PI3K complex then produces phosphatidylinositol 3-phosphate (PI3P) at the site of phagophore nucleation from the endoplasmic reticulum (ER), leading to the binding of PI3P-binding proteins, such as WD-repeat domain phosphoinositide-interacting protein 1 and 2 (WIPI/II). WIPI/II then recruits the elongation machinery to the phagophore, further facilitating phagophore maturation. 

##### LC3 Conjugation System and Cargo Loading

LC3-II is a proteolipid molecule that stably associates with phagophore membranes, where it facilitates phagophore elongation and closure. LC3-II is generated in a multistep process. First, ProLC3B is cleaved by the cysteine protease ATG4B to yield its soluble form LC3-I. LC3-I is then conjugated with phosphatidylethanolamine (PE) through ubiquitin-like conjugation systems that include the E1 ligase ATG7, E2 ligase ATG3, and E3 ligase complex, which is composed of ATG12, ATG5, and ATG16L. This PE-conjugated form of LC3, called LC3-II (shown as small green circles), is then incorporated into growing phagophore membranes, where it facilitates phagophore elongation and closure. LC3-II also serves as a docking site for several autophagy receptor proteins, such as SQSTM1/p62 and NBR1, which facilitate the delivery of autophagic cargo to the autophagosome. These autophagy cargo receptor proteins harbor LC3-interacting regions (LIRs) and bind to both LC3-II and its specific cargo, enabling a process known as selective autophagy.

##### Closure of the Phagophore Membrane and Fusion with the Lysosome

Once the cargo is loaded and enclosed by the phagophore membrane, it is called an autophagosome. Autophagosomes fuse with lysosomes to form autolysosomes. The trapped cargo is degraded by lysosomal enzymes, yielding nutrients that are recycled back into the cytosol for subsequent bioenergetic and anabolic processes. Thus, autophagy ensures the delivery, degradation, and recycling of intracellular substrates. 

##### Inhibitors of Autophagy

To date, several inhibitors have been developed that target the respective steps and molecules involved in the autophagy-lysosome pathway. These include inhibitors of ULK1/2 (SBI-0206965 [69], MRT68921, MRT 67307 [70], ULK101 [71]), VPS34 (VPS34-In1 [72], PIK-III [73], SAR405 [74], compound 31 [75], Spautin1 [76]), ATG4B (S130 [77], FMK-9a [78], NSC185058 [79]), and lysosomes (chloroquine [CQ]/hydroxychloroquine [HCQ], BafA1, Lys05 [80], ROC-305 [81], and GNS561 [82]) (Figure 3). 

#### 3.2.3. Autophagy Suppresses Tumor Initiation in the Pancreas

The functions and roles of autophagy in cancer are complicated and highly context-dependent; however, roughly speaking, the nutrient-recycling role of autophagy promotes the growth of established tumors by supplementing the metabolic demands of highly proliferative cancer cells, while the homeostatic role, mainly mediated by selective autophagy, prevents the development of cancer by removing oncoproteins or carcinogenic components from normal cells or precancerous cells [62]. 

Most evidence supporting the role of autophagy as a suppressor of tumorigenesis comes from studies using genetically engineered mouse models, where the loss of essential autophagy genes in specific organs enhanced carcinogenesis. For example, mosaic deletion of autophagy-related (Atg)-5 in the whole body or liver-specific deletion of Atg7 leads to the spontaneous formation of benign tumors (adenoma) in the mouse liver [83,84]. Similarly, in *KRAS*-driven lung cancer and PDAC models, loss of Atg5 or Atg7 in the lung or pancreas increases the number of benign lung tumors or precancerous lesions in the pancreas and pancreatic intraepithelial neoplasia (PanIN) [85,86,87,88,89]. In the absence of oncogenic KRAS, autophagy deletion alone does not lead to PanIN formation [88,89,90]. Importantly, these autophagy-deficient premalignant lesions remain benign and do not progress to a malignant state (i.e., cancer). Collectively, these results suggest a dual role for autophagy in carcinogenesis: blocking the initiation of preneoplastic lesions while promoting the progression of these benign lesions to invasive cancers.

This enhanced tumorigenesis upon autophagy ablation has been attributed to the accumulation of reactive oxygen species (ROS), DNA damage, and defective mitochondria, all of which are potentially carcinogenic [91]. Indeed, autophagy loss has been shown to induce genomic instability and aneuploidy [92,93]. Moreover, autophagy loss contributes to tumorigenesis by activating nuclear factor-erythroid 2-related factor 2 (NRF2) signaling, a central pathway regulating redox and stress response genes [94]. In the mouse liver, autophagy loss leads to the accumulation of autophagy cargo receptor protein sequestosome 1 (SQSTM1/p62), which is normally degraded via autophagy together with its substrates. Accumulated SQSTM1/p62 then traps Keap1, a negative regulator of NRF2, and releases NRF2 from Keap1-mediated regulation, leading to the NRF2 signaling activation and enhancing adenoma formation in the liver [84,95,96,97]. Indeed, SQSTM1/p62 accumulation is frequently observed in chronic liver diseases and hepatocellular carcinoma [84,97] in the form of Mallory–Denk bodies [98], further supporting the role of SQSTM1/p62 accumulation in liver tumorigenesis.

#### 3.2.4. Autophagy Promotes PDAC Progression in Metabolic-Dependent and -Independent Manners

In contrast to the context-dependent roles of autophagy in carcinogenesis, autophagy generally plays a pro-tumorigenic role in established cancers. Particularly, *RAS*- or *RAF*-driven cancers, such as PDAC, lung cancer, and melanoma, show constitutively high levels of basal autophagy, and autophagy inhibition slows tumor growth [36,85,86,88,99,100,101,102,103]. 

The crucial role of autophagy in maintaining PDAC growth has been demonstrated in a series of studies that included human PDAC cell lines, genetically engineered mouse models of PDAC, and patient-derived xenograft models. The first study showed that human PDAC tumors and cell lines exhibit elevated levels of basal autophagy and autophagy inhibition, either genetically or pharmacologically, with CQ, reduced tumor growth via impaired mitochondrial metabolism and ROS accumulation [101]. This was later confirmed in a genetically engineered mouse model of PDAC (Pdx1-Cre+; LSL-Kras^G12D/+^; Trp53^flox/+^), where biallelic loss of Atg5 reduced PDAC formation and improved mouse survival [88]. Atg5-null tumors exhibit impaired proliferation, increased DNA damage, and increased apoptosis [88]. Consistently, pharmacological inhibition of autophagy with the lysosomal inhibitor HCQ exerted tumor-suppressive effects in a panel of patient-derived PDAC xenografts, regardless of Trp53 status [88]. In a more recent study that developed a new genetically engineered mouse model of PDAC, where autophagy can be blocked in a doxycycline-inducible manner, it was demonstrated that autophagy inhibition in established PDAC tumors caused drastic tumor regression [89]. 

How does autophagy support cancer progression in established tumors? Because proliferative cancer cells require high amounts of metabolic intermediates to drive bioenergetic and anabolic processes, autophagy-mediated nutrient recycling significantly helps cancer cells meet their increased metabolic demands [104,105]. Indeed, autophagy-mediated nutrient recycling fuels the TCA cycle in cancer cells, and autophagy inhibition causes defects in mitochondrial respiration and ATP synthesis [85,86,88,99,101,106]. In PDAC, nutrient recycling and scavenging through autophagy and macropinocytosis are critical for maintaining the intracellular amino acids pool [107]. 

In addition to the nutrient recycling roles of autophagy, recent studies have also revealed the non-metabolic functions of autophagy, such as treatment resistance and immune evasion. Moreover, autophagy in normal host cells (host autophagy) and cancer cells have been shown to promote PDAC progression. This is discussed in the following sections.

#### 3.2.5. Mechanisms for the Enhanced Autophagy-Lysosomal Function in PDAC

As mentioned above, PDAC and several other cancers display constitutively activated autophagy-lysosomal functions irrespective of nutrient availability. Indeed, cell lines of these cancer types typically show elevated autophagy, even when grown in nutrient-rich culture media. Notably, these cancers have evolved ways to employ both catabolic processes mediated by the autophagy-lysosomal pathway and anabolic processes driven by mTORC1, enabling efficient nutrient recycling and cell proliferation.

**Figure 4:**
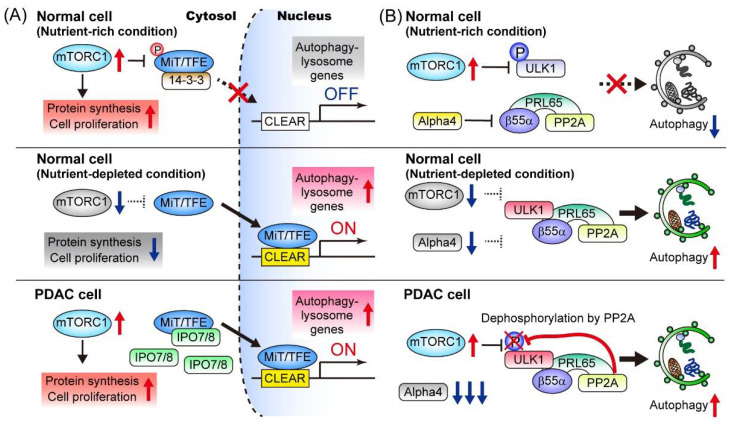
Mechanisms for the constitutive activation of autophagy in PDAC cells. (**A**) Under nutrient-rich condition, the mechanistic target of rapamycin complex 1 (mTORC1) phosphorylates microphthalmia (MiT)/transcription factor E (TFE), trapping these transcription factors in the cytosol. In PDAC cells, overexpression of IPO7 and IPO8 prevents mTORC1-mediated cytosolic retention of the MiT/TFE transcription factor [107]. (**B**) Under nutrient-rich condition, active mTORC1 phosphorylates ULK1 at S637, thus blocking ULK1-mediated autophagy initiation. In PDAC cells, the expression of Alpha4, a negative regulator of PP2A phosphatase activity, is downregulated. Thus, the PP2A-PRL64-β55α complex actively dephosphorylates S637 on ULK1, thereby counteracting mTORC1-mediated autophagy inhibition [114]. Note that both mechanisms allow PDAC cells to employ mTORC1-driven anabolic processes and autophagy-mediated nutrient recycling. Figure 4 is adapted from reference [41], the use of which is licensed under a Creative Commons Attribution 4.0 International license https://creativecommons.org/licenses/by/4.0/.

The initiation of autophagy is tightly regulated by the ULK1 complex, which integrates signals from mTORC1 and AMPK, the two major sensors of nutrient availability and cellular energy status. In addition to this kinase-dependent regulation of autophagy induction, transcriptional programs also play a role in maintaining enhanced autophagy-lysosomal function. The microphthalmia/transcription factor E (MiT/TFE) family of transcription factors (MITF, TFE3, and TFEB) regulates autophagy and lysosome biogenesis through the coordinated upregulation of autophagy- and lysosome-related genes [108,109,110]. Under nutrient-replete conditions, MiT/TFE proteins are phosphorylated by mTORC1, which leads to interactions with 14-3-3 proteins, a family of regulatory molecules, and cytoplasmic retention. Under starvation, mTORC1 is inactivated and MiT/TFE factors can translocate into the nucleus, where they activate the transcription of their target genes by directly binding to the coordinated lysosomal expression and regulation (CLEAR) sequence [109] (Figure 4). Perera et al. showed that MiT/TFE proteins are constitutively localized in the nucleus of PDAC cells, irrespective of their nutrient status [107]. Mechanistically, PDAC cells overexpress both MiT/TFE proteins and the nuclear import proteins importin (IPO)-7 and IPO8, thereby facilitating the nuclear translocation of MiT/TFE factors and sustaining the elevated autophagy-lysosomal function, though it remains to be elucidated how increased abundance of IPO7/8 decouples MiT/TFE factors from mTORC1-mediated regulation. Notably, ablation of MiT/TFE in PDAC cells significantly abrogated autophagy-lysosomal function and suppressed tumor growth, highlighting the crucial role of this transcriptional program in PDAC [107] (Figure 4).

As another mechanism for the sustained autophagy activation in PDAC, Wong et al. demonstrated that protein phosphatase 2A (PP2A) activity is increased in PDAC, which counteracts mTORC1-mediated autophagy suppression. Mechanistically, mTORC1 suppresses autophagy through the phosphorylation of ULK1 at multiple sites, including S637 and S757 [111,112] (Figure 3), though in PDAC the PP2A–PRL65-B55α complex actively dephosphorylates S637 on ULK1, thereby counteracting this mTORC1-mediated autophagy suppression while exploiting other mTORC1-driven anabolic processes to facilitate cell proliferation (Figure 4). 

How do PDAC cells maintain lysosomal membrane integrity while coping with a massive cargo influx? Using a proteomics-based approach, Gupta et al. identified that the plasma membrane repair factor Myoferlin is enriched in the lysosomal membranes of PDAC cells, where Myoferlin helps maintain lysosomal membrane integrity and function [113]. Indeed, Myoferlin expression is increased in human PDAC tumors and is associated with a shorter patient prognosis. Notably, genetic knockout of Myoferlin impairs tumor growth in murine PDAC models, highlighting its critical role in PDAC biology [113].

#### 3.2.6. Autophagy Is a Driver of Treatment Resistance

Autophagy has been implicated in resistance [115,116,117]. Notably, recent studies have identified autophagy as a mechanism of resistance to inhibition of the mitogen-activated protein kinase (MAPK) pathway, a major downstream pathway of oncogenic KRAS. This was initially observed in a mouse model of PDAC, where genetic ablation of KRAS or pharmacological inhibition of the MAPK pathway led to rapid tumor shrinkage, while a small population of PDAC cells survived. Interestingly, surviving PDAC cells exhibit elevated autophagic flux, which is indispensable for their survival [118]. In line with this, several recent studies have demonstrated that autophagy flux is increased upon KRAS or MAPK inhibition in PDAC or other KRAS or RAF-driven cancers, and that the combined inhibition of autophagy and the MAPK pathway exerts potent anti-tumor activity in a synergistic manner [119,120,121]. Notably, a more recent study showed that combination therapy using the MAPK pathway inhibitor cobimetinib and the lysosomal inhibitor mefloquine, but not either treatment alone, activates the STING/type I interferon pathway in PDAC cells, which in turn switches TAMs into a tumor-suppressive M1-like phenotype [122]. This immunological change is further augmented by the addition of an agonistic antibody to CD40, a costimulatory molecule that licenses and activates antigen-presenting cells upon ligand engagement [123], inducing a robust T-cell response [122]. Based on these promising preclinical findings, multiple clinical trials are currently testing the efficacy of a combined treatment with HCQ and MEK/ERK inhibitors (Table 1).

#### 3.2.7. Autophagy Facilitates Immune Evasion in PDAC

PDAC is known for its resistance to ICB. This has long been attributed to multiple factors, including immune suppressive TME, paucity of tumor-infiltrating CD8^+^ T cells, and lack of neoantigens [27], all of which have been well appreciated in genetically engineered models of PDAC [124]. However, more recent evidence has demonstrated that human PDAC tumors have a varying number of CD8^+^ T cells with considerable spatial heterogeneity within the tumor [125,126,127] and that some patients with PDAC actually harbor neoantigens that can drive potent T cell immunity [18,128,129], suggesting the existence of other undiscovered factors that drive resistance to ICB. 

Major histocompatibility complex class I (MHC-I) protein is a critical component of the acquired immune system. CD8^+^ T cells recognize and kill cancer cells that present neoantigens via MHC-I on their cell membranes. Unsurprisingly, loss of MHC-I is frequently observed across cancer types and is associated with a poor response to ICB [130,131,132,133]. MHC-I expression is downregulated in both human and mouse PDAC tumors, which is particularly evident in liver metastases [134,135]. However, unlike other types of cancer, MHC-I mutations are rare in human PDAC [16]. 

**Figure 5:**
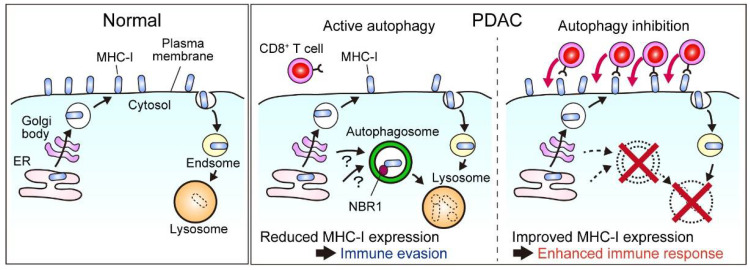
Autophagy facilitates immune evasion of PDAC via the selective removal of the major histocompatibility complex class I (MHC-I) [136,137]. In PDAC cells, MHC-I is degraded via NBR1-mediated selective autophagy, thus reducing the cell surface MHC-I expression and promoting the immune evasion of PDAC cells. Blocking autophagy or lysosome restores the cell surface MHC-I levels in PDAC cells and improves CD8^+^ T cell-mediated PDAC cell recognition and killing, triggering a robust anti-tumor immune response that can be further augmented via combined treatment with immune checkpoint blockade (ICB) agents. Figure 5 is adapted from reference [41], the use of which is licensed under a Creative Commons Attribution 4.0 International license https://creativecommons.org/licenses/by/4.0/.

A recent study demonstrated that MHC-I is actively degraded through an autophagy-dependent mechanism, leading to decreased levels of cell surface MHC-I expression in PDAC cells, which helps PDAC cells to escape immune surveillance. Mechanistically, this requires an autophagy cargo receptor protein, NBR1, which binds to polyubiquitinated MHC-I molecules through its ubiquitin-associated domain and recruits them to the autophagosome, enabling selective removal of MHC-I in PDAC. Importantly, genetic or pharmacological inhibition of autophagy restores cell surface MHC-I expression and antigen presentation in PDAC cells, leading to increased CD8^+^ T cell infiltration, reduced tumor burden, and improved response to ICB in syngeneic tumor transplant mouse models [136,137] (Figure 5). Consistently, a recent clinical trial demonstrated that the addition of HCQ to gemcitabine plus nab-paclitaxel (GnP), a standard-of-care chemotherapy for PDAC [6], in the context of neoadjuvant chemotherapy leads to increased CD8^+^ T cell infiltration in resected tumors [138,139]. In line with these findings, a recent study identified cancer cell-derived progranulin (PGRN) as a driver of autophagy-dependent MHC-I degradation and subsequent immune evasion [140]. Using surgically resected human PDAC specimens, the authors showed that increased PGRN expression in PDAC cells was associated with reduced MHC-I expression, decreased CD8^+^ T cell infiltration, and worse patient prognosis. Notably, blocking PGRN with a neutralizing antibody abrogated autophagy-mediated MHC-I degradation and restored MHC-I expression in PDAC cells, improving anti-tumor T cell responses in mouse PDAC models [140]. Similarly, unbiased studies using CRISPR-mediated knockout screens in vitro and in vivo have identified autophagy-related genes as critical factors to escape CD8^+^ T cell-mediated killing [141,142]. Together, these studies highlight autophagy as a promising target to sensitize PDAC to immune therapy, warranting further studies to better understand the underlying mechanisms and develop novel combination regimens for PDAC treatment. 

#### 3.2.8. Autophagy Is a Negative Regulator of the Anti-Tumor Immune Response in Cancer

In addition to PDAC, autophagy contributes to immune evasion in various cancer types. Notably, in multiple unbiased screening studies using genome-wide CRISPR knockout libraries, autophagy has been identified as a key driver of immune evasion across cancer types [143,144,145]. The major mechanisms include impaired antigen presentation [136,137,140,146], downregulation of T cell-recruiting chemokines [147,148,149,150], and resistance to CD8^+^ T cell-mediated killing [143,144,145]. Besides cancer cell autonomous autophagy, autophagy-related processes in non-cancerous host cells also promote tumor immune evasion [151,152,153,154]. Based on these compelling preclinical findings, multiple clinical trials are [155] currently ongoing to test the combined blockade of autophagy and immune checkpoint molecules in PDAC (Table 1) and other malignancies.

#### 3.2.9. Host Autophagy Supports Tumor Growth

In addition to autophagy in cancer cells, autophagy in normal host cells, termed host autophagy, has been shown to promote cancer progression. This was discovered in a series of novel mouse models of lung cancer, melanoma, and PDAC, where autophagy can be inhibited separately in host cells or cancer cells in a tamoxifen- or doxycycline-dependent manner [89,102,154,156]. In these models, autophagy inhibition in normal host cells unexpectedly slowed the growth of autophagy-competent tumors, shedding light on the role of host autophagy. Mechanistically, host autophagy promotes cancer progression by providing nutrients or promoting immune tolerance.

##### Host Autophagy Provides Nutrients

Pancreatic stellate cells (PSCs) are a major precursor of CAFs in PDAC tumors [157]. Sousa et al. demonstrated that autophagy is activated in PSCs and PDAC cells in pancreatic tumors. Notably, PSCs secrete alanine, a non-essential amino acid, in an autophagy-dependent manner. This PSC-derived alanine is taken up by PDAC cells, where alanine fuels the tricarboxylic acid cycle for de novo synthesis of other non-essential amino acids and free fatty acids (Figure 1, green). Consequently, PDAC can utilize glucose and glutamine, two critical nutrients that are depleted in the PDAC TME [33,34], for other anabolic pathways, including nucleic acid production [35] or NADPH production to maintain redox balance [45], enabling unique metabolic rewiring in PDAC (Figure 1). A follow-up study by the same group identified critical amino acid transporters involved in this process: PSCs upregulate SLC1A4 expression to secrete alanine, while PDAC cells upregulate SLC38A2 to take up alanine. Notably, genetic ablation of SLC38A2 causes a serious metabolic crisis in PDAC cells, but not in normal pancreatic cells, leading to tumor regression in mouse models of PDAC [158]. These results confirm the critical role of PSC-secreted alanine in PDAC metabolism, representing a clinically relevant vulnerability to this deadly disease. 

In addition to alanine, PSCs/CAFs secrete multiple metabolites, cytokines, chemokines, and growth factors, thus contributing to the formation of a complicated TME in PDAC tumor [159,160,161]. Notably, autophagy is required for the activation of PSCs into CAFs to acquire tumor-promoting properties [155,162] along with other stimuli.

Host autophagy has also been shown to support tumor metabolism in other types of cancer. Using a murine melanoma model, it has been shown that melanoma cells that require exogenous arginine for survival are particularly vulnerable to the loss of host autophagy. Mechanistically, autophagy inhibition in hepatocytes increased circulating arginase I, an arginine-degrading enzyme, thereby reducing serum arginine levels and the growth of arginine auxotrophic tumors [156]. What are the mechanisms underlying autophagy induction in host cells? In an *RAS*-driven cancer model in *D. melanogaster*, tumor-secreted ROS have been shown to induce autophagy in host cells, which leads to amino acid secretion from host cells and subsequently promotes tumor growth [163].

##### Host Autophagy Promotes Immune Tolerance 

Recent studies have also demonstrated that the loss of autophagy in non-cancerous host cells leads to enhanced anti-tumor immune responses across cancer types, shedding light on the role of host autophagy as a negative regulator of anti-tumor immunity.

Loss of autophagy in CD8^+^ T cells enhances their tumor cell-killing function, leading to impaired tumor growth in murine tumor allograft models [153]. Mechanistically, autophagy loss shifts CD8^+^ T cells towards a more glycolytic state, leading to increased production of S-adenosylmethionine, an important methyl-donor molecule, and subsequent alterations in the epigenetic landscape. Consequently, CD8^+^ T cells are transcriptionally reprogrammed to an effector memory state that can efficiently kill cancer cells. 

In murine melanoma allograft models with a high mutational burden, loss of host autophagy increases tumor-infiltrating T cells and impairs tumor growth in a T cell-dependent manner [154]. Mechanistically, host autophagy, particularly autophagy in the liver, negatively regulates anti-tumor T cell responses, in part by enhancing the function of regulatory T cells (Tregs) and suppressing interferon-γ signaling. 

Similarly, other autophagy-related pathways in host cells are also involved in the regulation of anti-tumor immune responses. LC3-associated phagocytosis (LAP) is a form of phagocytosis that uses several core autophagy components, including VPS34, BECN1, ATG3, ATG5, ATG7, and ATG12. Macrophages use LAP to clear pathogens or dying cells, which is an essential process for determining subsequent immune responses. Indeed, the impairment of LAP in immune cells leads to autoimmune disease-like states [164]. In the context of anti-tumor immunity, loss of LAP, but not autophagy, reprograms macrophages from the tumor-promoting M2 subtype to the tumor-suppressive M1 subtype, thus enhancing anti-tumor T cell responses and blocking tumor growth [151]. Additionally, the lysosomal inhibitor chloroquine (CQ), an anti-malarial agent that has frequently been used to inhibit autophagy in various clinical trials, has been shown to directly reprogram macrophages from the M2 to M1 phenotype, thus enhancing anti-tumor T cell responses [152]. 

Together, these studies highlight the roles of host autophagy and autophagy-related processes as critical mediators of anti-tumor immune responses. These results indicate that inhibiting autophagy systemically in the whole body might be more efficacious than inhibiting autophagy specifically in cancer cells, although such inhibitors have yet to be developed.

#### 3.2.10. Clinical Trials Targeting Autophagy

Based on these basic findings, numerous clinical trials have been conducted to assess the therapeutic benefits of autophagy inhibition in PDAC. The first single-arm phase II study that used HCQ as monotherapy in third-line chemotherapy for patients with advanced PDAC failed to show objective responses [165]. This is likely because these patients may not have received sufficient HCQ because they already experienced disease progression after multiple series of treatments and were unable to receive HCQ for a long period. However, more recent studies that used HCQ in combination with potent chemotherapy have shown promising results. A phase I/II trial was conducted to assess the efficacy of HCQ plus gemcitabine as preoperative treatment for patients with borderline resectable PDAC. The combination treatment was well tolerated and the curative resection rate was as high as 70%, which is a better result than the historical control in the same institute [166]. Based on this result, a randomized phase II trial was conducted to assess the efficacy of HCQ plus GnP, the current standard-of-care treatment for PDAC, in previously untreated advanced-stage PDAC. Although the addition of HCQ to GnP failed to increase progression-free survival (PFS) or overall survival (OS), the response rate was significantly higher in patients receiving HCQ plus GnP than in those receiving GnP alone (21% vs. 38%, *p* = 0.047) [167]. In 2020, another randomized phase II trial was conducted to compare the efficacy of HCQ plus GnP and GnP alone in preoperative chemotherapy. An analysis of resected tumor specimens demonstrated an improved pathological response and increased tumor-infiltrating immune cells, including CD8^+^ T cells [138,139] (NCT01978184), supporting the role of autophagy as a driver of immune escape in PDAC [136,137]. Based on these findings, multiple clinical trials are ongoing to test the therapeutic efficacy of combining HCQ with conventional chemotherapy, molecular-targeted therapy, and ICB (Table 1).

It should be noted that these studies used the lysosomal inhibitor HCQ to block autophagy because there are currently no autophagy-specific inhibitors approved for clinical use. Moreover, HCQ has long been used in the treatment of patients with malaria infection or autoimmune diseases, such as systemic lupus erythematosus, and its safety is well described. In contrast to compelling preclinical evidence showing the efficacy of autophagy inhibition in PDAC, the impact of HCQ on patient outcomes in clinical trials may be modest. This may be explained in part by the unfavorable pharmacokinetics of HCQ in vivo [168], highlighting the need to develop more potent inhibitors. Along this line, it is also unknown whether selective blocking of autophagy is better than blocking all lysosome-related processes, such as autophagy, using HCQ. 

#### 3.2.11. Resistance to Autophagy Inhibition

When considering autophagy inhibition as a therapeutic strategy, the emergence of resistance is an inevitable obstacle. Upregulation of Nrf2 signaling, a key transcription factor that regulates cellular stress responses [94], has been identified as a mechanism of autophagy inhibition resistance in cancer cells [169,170]. Under physiological conditions, Nrf2 is constitutively degraded by Keap1 via the UPS. In autophagy-deficient cells, the autophagy cargo receptor protein p62, which is normally degraded through the autophagy-lysosomal pathway, together with its autophagy cargo, accumulates in the cytosol. Accumulated p62 releases Nrf2 from Keap1-mediated degradation [95,96,171], derepressing Nrf2 function [169,170]. Enhanced Nrf2-pathway and activation of downstream anti-stress programs allow cancer cells to cope with ER stress induced by autophagy loss [169]. Moreover, enhanced Nrf2 signaling upregulates the transcription of macropinocytosis-related genes, thereby increasing extracellular nutrient scavenging to compensate for the loss of intracellular scavenging via autophagy [170]. Indeed, blocking these compensatory mechanisms abrogates resistance to autophagy inhibition in PDAC cells [169,170], suggesting that this is a potential therapeutic approach for this cancer. 

## 4. Metabolic Crosstalk between PDAC and Stromal Cells

PDAC tumors are characterized by prominent stromal expansion, which is composed of numerous non-neoplastic cells, including PSCs, CAFs, nerve cells, and immune cells, all of which play both tumor-promoting and tumor-suppressing roles. Recent studies have demonstrated that these stromal cells actively interact with cancer cells by exchanging metabolites, which not only supports PDAC metabolism but also confers therapeutic resistance. 

### 4.1. PSCs/CAFs

PSCs/CAFs are the most abundant cell type in the TME of PDAC and secrete multiple cytokines and growth factors, adding complexity to the PDAC TME [157]. In addition to these soluble mediators, PSCs/CAFs also secrete various metabolites that have significant effects on PDAC metabolism, progression, and therapeutic resistance.

As described in the section on host autophagy, PSCs secrete several amino acids, including alanine, in an autophagy-dependent manner. PSC-derived alanine is taken up by PDAC cells and utilized to produce other amino acids and lipids, enabling PDAC cells to save glucose and glutamine for other biosynthetic pathways [155,158]. What are the sources of these PSC-derived amino acids? Zhang et al. demonstrated that CAFs actively scavenge extracellular proteins via macropinocytosis, which yields amino acids that are eventually secreted to fuel PDAC metabolism [172]. These findings suggest the potential merit of using CQ/HCQ, a lysosomal inhibitor that can block both autophagy and other lysosome-related pathways, including macropinocytosis, for PDAC treatment. However, it should be noted that CQ/HCQ has recently been shown to have only a weak inhibitory effect on macropinocytosis [170], highlighting the need for developing selective inhibitors of macropinocytosis. 

During activation by PSCs, CAFs undergo drastic changes in lipid metabolism, lose their lipid droplets, and secrete abundant lipids [173]. Auciello et al. demonstrated that among the CAF-secreted lipids, lysophosphatidylcholines are utilized by PDAC cells to synthesize phosphatidylcholines and lysophosphatidic acid, which are key components of cell membranes and potent mediators of the wound healing process, thereby promoting PDAC proliferation and migration [173]. 

PSC/CAF-derived metabolites also promote therapeutic resistance in PDAC cells. For example, CAFs secrete nucleotides, including deoxycytidine, which blunts the efficacy of the nucleotide analog gemcitabine and confers therapeutic resistance [174]. 

### 4.2. Neurons

PDAC is well known for its frequent perineural invasion, which is the major cause of severe persistent pain in PDAC patients [175,176]. Importantly, surgical or pharmacological ablation of nerves prolongs the survival of tumor-bearing animals [177,178], supporting the tumor-promotive role of nerves in PDAC. More specifically, sympathetic nerves promote PDAC progression in part via catecholamines, which activate the adrenergic β-2 receptor (ADRB2) on PDAC cells and promote PDAC proliferation [178], while cholinergic nerves suppress PDAC growth via cholinergic signaling, which inhibits the MAPK pathways in PDAC cells [179]. 

In addition to these neurotransmitters, a recent study revealed that peripheral nerve cells also secrete amino acids, such as serine, which support PDAC metabolism and growth [180]. Serine is a non-essential amino acid that can be synthesized from glucose via the serine biosynthesis pathway (SBP). Importantly, up to 40% of human PDAC tumors and cell lines lack key enzymes in SBP and cannot produce serine on their own; thus, they are completely dependent on exogenous serine for tumor growth. Indeed, nerve cell-secreted serine significantly supported the growth of SBP-deficient PDAC cells. Notably, SBP-deficient PDAC cells have an inherent program to promote tumor innervation via increased secretion of the nerve growth factor (NGF). Mechanistically, serine deprivation decreases the mRNA translation of TCC and TCT serine codons, leading to a preferential increase in *NGF* mRNA translation and NGF secretion. PDAC tumors lacking SBP tend to have higher levels of innervation, reflecting an increased dependence on neuronal serine support [180]. 

Notably, antagonizing ADRB2 or blocking TRK1, a receptor for NGF, significantly delays the tumor growth in murine models [178,180]. This is clinically relevant, as ADRB2 and TRK1 can be blocked with β-blockers, drugs used for the treatment of hypertension, and entrectinib, an inhibitor of TRK1 that is currently used for malignancies carrying *NTRK* gene fusions [23].

### 4.3. Macrophages

TAMs, one of the most abundant immune cell populations in the tumor stroma of PDAC [126], are primarily involved in the immune suppression and treatment resistance of PDAC. Indeed, the abundance of TAMs is associated with a poor response to treatment [181]. As a mechanism to drive chemoresistance, Halbrook et al. showed that TAMs release nucleic acid deoxycytidine, which antagonizes and confers resistance to gemcitabine in PDAC cells. Accordingly, depletion of TAMs, either genetically or pharmacologically, sensitizes PDAC cells to gemcitabine in murine PDAC models [182]. 

## 5. Conclusions

PDAC is characterized by distinct metabolic features that are driven by intrinsic cancer cell pathways as well as extrinsic cues imposed by the host cells. Each of these features represents an attractive therapeutic target; however, recent studies have shown that targeting a single pathway/mechanism is rarely sufficient, highlighting the extreme plasticity and flexibility of PDAC metabolism [47]. One possible way to overcome this is to co-target compensatory pathways, as exemplified by the combination of the RAS–MAPK pathway inhibitors and the lysosomal inhibitor, HCQ [119,120,121], or via the combination of autophagy and compensatory upregulation of macropinocytosis [170]. Notably, modulating tumor metabolism, which alone may not be sufficiently potent to block tumor progression, sometimes has an immense impact on the TME, including the anti-tumor immune responses, which can be synergistic with other modes of therapies, such as ICB [50,136,137,140,141,142]. In addition, targeting the metabolism in host cells in the TME, rather than PDAC cells, is an attractive approach to improve the therapeutic responses to other cancer-directed treatments, such as conventional chemotherapy [174,182] and ICB [151,153,154]. Therefore, continuous efforts to understand the metabolic features and vulnerabilities of PDAC will aid in the development of effective therapeutic approaches against this deadly disease.

## Figures and Tables

**Table 1:** Ongoing clinical trials targeting autophagy/lysosome in pancreatic ductal adenocarcinoma (PDAC).

Disease	Trial Phase	Therapy	NCT No.
Metastatic	I	GnP + HCQ + Ipilimumab (anti-CTLA4 antibody)	NCT04787991
Metastatic	I/II	Cobimetinib (MEK inhibitor) + HCQ + Atezolizumab (anti-PDL1 antibody)	NCT04214418
Unresectable	I	Trametinib (MEK inhibitor) + HCQ	NCT03825289
Metastatic	I	Binimetinib (MEK inhibitor) + HCQ	NCT04132505
Unresectable	I	Ulixertinib (ERK inhibitor) + HCQ	NCT04145297
Metastatic	II	LY3214996 (ERK inhibitor) + HCQ	NCT04386057
Unresectable	I	mFOLFIRINOX + HCQ + Chlorphenesin Carbamate	NCT05083780
Unresectable	II	GnP + HCQ + Paricalcitol (Vitamin D receptor agonist)	NCT04524702
Resectable (neoadjuvant)	I	HCQ + Paricalcitol (Vitamin D receptor agonist) + Losartan. Used after neoadjuvant mFOLFIRINOX + RT and prior to surgery.	NCT05365893
Unresectable and borderline resectable	II	Gem + Cisplatin + Paclitaxel protein bound + HCQ	NCT04669197
Resectable (neoadjuvant)	I/II	mFOLFIRINOX + HCQ	NCT04911816

GnP, gemcitabine + nab-paclitaxel; RT, radiation therapy.
